# The effect of pulp inflammation and premature extraction of primary molars on the successor permanent teeth. A retrospective study

**DOI:** 10.1111/ipd.12568

**Published:** 2019-09-18

**Authors:** Fawn Nitanee van der Weijden, Daniela Hesse, Gabriela Caldeira Andrade Americano, Vera Mendes Soviero, Clarissa Calil Bonifacio

**Affiliations:** ^1^ Department of Orthodontics Academic Centre of Dentistry Amsterdam Amsterdam The Netherlands; ^2^ Department of Paediatric Dentistry Academic Centre for Dentistry Amsterdam Amsterdam The Netherlands; ^3^ Department of Paediatric Dentistry Universidade do Estado do Rio de Janeiro Rio de Janeiro Brazil; ^4^ Dental School, Faculdade Arthur Sá Earp Neto Petrópolis Brazil

**Keywords:** dental arch, developmental enamel defects, malocclusion, permanent dentition, primary tooth, pulpitis, tooth extraction

## Abstract

**Background:**

Untreated caries on primary molars often leads to pulp inflammation and extraction.

**Aim:**

To retrospectively investigate the effect of pulp inflammation and extraction of primary molars on their successors regarding alignment in the dental arch and developmental enamel defects (DED).

**Design:**

The participants in this study were children at public schools in Petropolis (Brazil), who participated in a 3‐year longitudinal clinical trial. Children (N = 44) were selected for the present study if they had at least one erupted premolar of which the predecessor primary molar presented pulp inflammation at baseline or during any of the 6‐month follow‐up assessments. All premolars were examined for DED and misalignment. Distinction was made between extraction performed before (E <8) or after the age of 8 years (E ≥8). Distinction was also made between pulp inflammation occurred before (*P* < 7) or after the age of 7 years (*P* ≥ 7). A logistic regression analysis was performed, and the odds ratio was calculated.

**Results and conclusions:**

Misalignment occurred more frequently in E <8 as compared to E ≥8 (OR = 2.85; *P* = .03). There was no significant difference in DED between *P* < 7 and *P* ≥ 7.

**Conclusion:**

Misalignment of premolars occurs more frequently when the predecessor primary molars are extracted before the age of 8 years.

## 1 INTRODUCTION

Although dramatic improvements in caries prevalence and severity have occurred, in Brazil there are still a persistent high number of children with untreated caries^1^ and early tooth loss.^2^ Untreated caries and early tooth loss have a negative impact on the oral health‐related quality of life of children, especially regarding oral pain and functional limitations domains.^2^, ^3^

Pulp inflammation due to untreated caries can lead to early loss of primary molars, either by early exfoliation (due to abscess formation and alveolar bone destruction) or by early extraction. In the first case, it is reported that about 90% of the successors erupt without space loss in the premolar area.^4^ In the latter case, however, the incidence of space loss is reported to be high, with at least 96% of extraction diastemas showing some space loss when present for more than 12 months.^5^

Although premature loss of primary molars can lead to migration of neighbouring teeth and lack of space for their successors, the moment when this premature loss occurs may determine its effect on the alignment of successor premolars in the dental arch.^6^ Generally, the earlier the extraction is performed, the greater the closure of the diastema.^5^ This holds particularly for extractions performed before the eruption of first permanent molars.^5^, ^7^ Furthermore, extraction of primary molars before the age of 8 years has been found to delay the eruption of permanent successors.^8^ Therefore, the age of 8 years could be a good cut‐off point to predict whether or not misalignment after extraction can be expected; however, the literature lacks on studies to clarify this issue.

Apart from early tooth loss, the presence of caries^9^, ^10^ and pulp inflammation^11^ on primary teeth is also associated with the occurrence of developmental enamel defects (DED) on the successor permanent teeth. In fact, Lo et al^9^ verified that the severity of the caries lesion on a primary molar can influence the presence of DED on the successor permanent tooth, and the authors concluded that the larger and deeper the caries lesion in a primary tooth is (that often required pulp therapy or extraction), the higher the chances are that the permanent successor tooth is affected by DED. According to the literature, the crown of premolars is completely formed and mineralized at the age of 5‐6 years (first premolar) and 6‐7 years (second premolar).^12^, ^13^ Thus, one could hypothesize that the crown of an unerupted premolar would be less susceptible to the consequences of an inflammation on the predecessor primary molar in children aged 7 years and older. To date however, there are no studies to support such a presumption.

Therefore, the purpose of this study was to evaluate in a group of school children, if there is a difference in alignment of premolars in the dental arch for those of which (a) the predecessor primary molar was extracted when the child was 8 years or older as compared to (b) the ones of which the predecessor primary molar was extracted before the child was 8 years old or to (c) those which have exfoliated naturally. Furthermore, this research investigated if there is a difference in occurrence of DED between premolars of which (a) the predecessor primary molar presented signs or symptoms of pulp inflammation when the child was 7 years or older as compared to (b) those of which the predecessor primary molar presented signs or symptoms of pulp inflammation before the child was 7 years old or to (c) those that never had any signs or symptoms of pulp inflammation.

## 2 MATERIALS AND METHODS

### 2.1 Protocol

The recommendations for strengthening the reporting were followed in accordance with the STROBE statement.^14^

### 2.2 Study design and ethics

The present study is a secondary analysis of data from another parallel randomized clinical trial conducted at public primary schools in Petropolis, a city in the state of Rio de Janeiro, Brazil. The socio‐economic status of the investigated population is low, and most had never visited a dentist before the start of the study. The main study received approval from the Human Research Ethics Committee of the Universidade Estatual do Rio de Janeiro (CAAE 20 592 113.5.0000.5259) and was registered at the Brazilian Trial Register (protocol number RBR‐3vzvbx). Only children who had a written consent from parents/caregivers were included in the study.

The main longitudinal study started in 2013 and aimed to compare two protocols for treating primary molars with multi‐surface cavitated caries lesions in high‐caries‐risk children, aged 4‐10 (mean 6.92; SD 1.58). One tooth per child was treated with either atraumatic restorative treatment (ART) or non‐restorative approach (NRA) by two trained operators, who were final‐year dental students.

Follow‐up evaluations were performed every 6 months during a period up to 36 months. The children were examined in a classroom in a supine position on a table, with the aid of a headlight, dental mirror, and sickle probe. During each follow‐up evaluation, a full intraoral exam was performed and signs or symptoms of pulp inflammation were assessed according to the PUFA index^15^ (Table 1). Also, in cases that the child had other treatments needs, dental care was provided by previous examiners that were involved in the ART or NRA protocol, and extractions were also performed when deemed necessary by those that provided the treatment.

**Table 1:** Criteria of the PUFA index^15^

Criteria	Description
P	Pulpal involvement is recorded when the opening of the pulp chamber is visible or when the coronal tooth structures have been destroyed by the carious process and only roots or root fragments are left. No probing is performed to diagnose pulpal involvement.
U	Ulceration due to trauma from sharp pieces of tooth is recorded when sharp edges of a dislocated tooth with pulpal involvement or root fragments have caused traumatic ulceration of the surrounding soft tissues, eg, tongue or buccal mucosa.
F	Fistula is scored when a pus‐releasing sinus tract related to a tooth with pulpal involvement is present.
A	Abscess is scored when a pus‐containing swelling related to a tooth with pulpal involvement is present.

### 2.3 Inclusion criteria and evaluations

**Figure 1:**
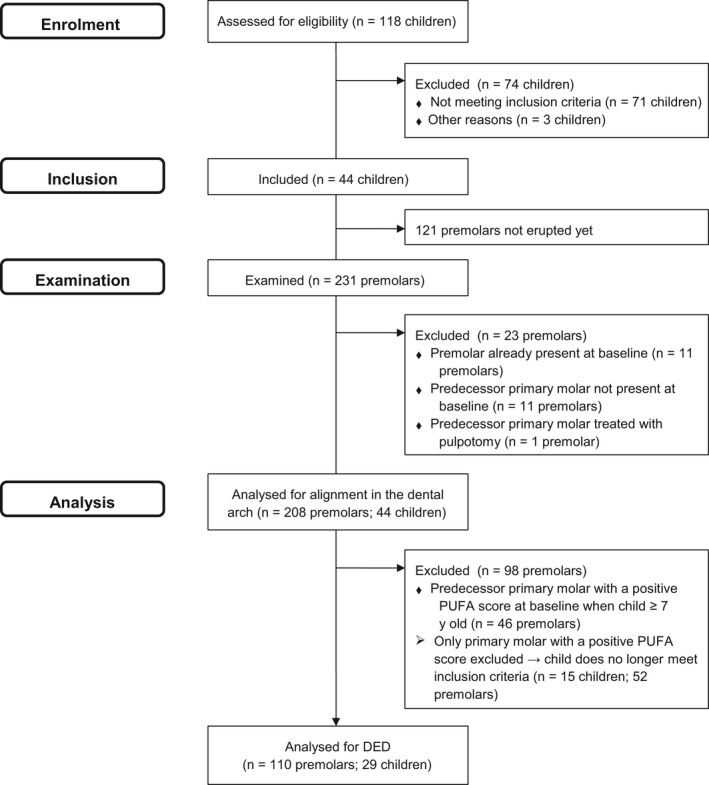
Flow diagram showing inclusion and exclusion of participants in the investigation

After 3 years from the beginning of the main study, all patients included in the main research were evaluated regarding the criteria described above. For the present investigation, only the children who had at least one erupted premolar (first/second, upper/lower) of which the predecessor primary molar presented signs or symptoms of pulp inflammation at baseline or any of the follow‐up evaluations were included (Figure 1). Those children were evaluated regarding the alignment of the premolars in the dental arch and the presence of DED. Before examination, supra‐gingival plaque was removed with a manual toothbrush and each tooth was dried with cotton rolls. The following evaluations were performed:

#### 2.3.1 Evaluation of alignment in the dental arch

Alignment of premolars in the dental arch was scored on a binary scale of having good alignment: ‘yes’ or ‘no’. This was scored by assessing the position of the premolar in relation to Angle's line of occlusion.^16^, ^17^ Any rotation, angulation, inclination, or deviation in buccal or lingual position was scored as misalignment. This evaluation was performed by two final‐year dental students (FNW and NAR), who were blinded to the dental history of the predecessor primary molar (extraction or physiological exfoliation). Clear deviations from Angle's line of occlusion were scored by one examiner only, and for smaller deviations, the judgement of the other examiner was consulted. Disagreements were solved after discussion with a ‘benchmark examiner’ (VMS).

#### 2.3.2 Evaluation of DED

The presence of DED was recorded according to the DDE index for general epidemiological surveys of the Fédération Dentaire International^18^ (Table 2). Distinction was made between diffuse and demarcated opacities. Because diffuse opacities are mainly due to fluorosis,^19^ only demarcated opacities were scored as DED. When the contralateral premolar showed a similar demarcated opacity, it was not recorded as DED. Furthermore, differentiation was done between DED and enamel caries lesions that occurred at a site with plaque accumulation (eg around the gingival margins). If hypoplasia was present, this was also noted. Examinations were performed by the same two dental students (FNW and NAR), who were blinded to the dental history of the predecessor primary molar (pulp inflammation). They were trained and calibrated by an experienced examiner (VMS). The examiners underwent a total of 5 hours of specific training and calibration session using pictures of clinical cases with fluorosis and demarcated opacities. The calibration exercise included a range of pictures, each of the two examiners had to score separately (sound, demarcated opacity or diffuse opacity/fluorosis). Subsequently, a hands‐on training including the evaluation of 8 children with DED on permanent teeth was performed. After two weeks, the same pictures were evaluated to calculate the intra‐examiner reliability. Disagreements during the evaluation of patients in this study were solved after discussion with the ‘benchmark examiner’ (VMS).

**Table 2:** Developmental enamel defect index for general epidemiological surveys of the Fédération Dentaire International^18^

Criteria	Description
Diffuse opacity	Opacity with poorly defined boundary, which merges into the surrounding enamel
Demarcated opacity	Opacity with clearly defined boundary from adjacent enamel
Hypoplasia	Quantitative defect in enamel, reduced thickness of enamel

### 2.4 Statistical analysis and exclusion criteria

All premolars of the selected children were examined for the alignment in the dental arch and presence of DED. For the analysis, premolars were excluded if they were already present at baseline or when the predecessor primary molar was not present at baseline, because in both these cases the status of the primary molar was unknown. Also, premolars of which the predecessor primary molar was treated with pulpotomy were excluded (Figure 1). The premolars of which the predecessor primary molars did not present signs or symptoms of pulp inflammation at baseline or during follow‐up were used as a control.

In addition, for analysis of DED, premolars were excluded in case the predecessor primary molar presented signs or symptoms of pulp inflammation at baseline when the child was 7 years or older (Figure 1). In these cases, it could not be ruled out that this pulp inflammation was already present before the age of 7.

For the outcome alignment of premolars in the dental arch, the following independent variables were tested: the age of children when the predecessor primary molar was extracted (Group E ≥8:8 years or older/Group E < 8: younger than 8 years/Group E0: exfoliated naturally), premolar (first/second), jaw (upper/lower), and sex (female/male). For the outcome occurrence of DED on premolars, the following independent variables were tested: age of children when pulp inflammation was detected (Group *P* ≥ 7:7 years or older/Group *P* < 7: younger than 7 years/ Group P0: never had any signs or symptoms of pulp inflammation), premolar (first/second), jaw (upper/lower), and sex (female/male). In both cases, a logistic regression analysis was performed and the odds ratio was calculated. P‐values < .05 were considered to be significant. The Cohen's Kappa was used to calculate the inter‐ and intra‐examiner reproducibility. The Cramer's V was used to calculate the effect size.

Data were organized using Microsoft Excel, and statistical analysis was carried out using the Statistical Package for Social Science (IBM SPSS Statistics 24). Additionally, G‐Power 3.0.10 was used for ‘post hoc’ power calculation.

## 3 RESULTS

### 3.1 Extraction and misalignment

#### 3.1.1 Descriptive

For the analysis of alignment in the dental arch, 44 children with 208 premolars could be included (Figure 1). These children (16 boys and 28 girls) had a mean age of 7.05 years (SD 1.22; range 4‐9) at baseline and 9.95 years (SD 1.22; range 7‐12) when the premolars were examined.

Out of the 208 predecessor primary molars, 131 (63%) had exfoliated naturally and 77 (37%) had been extracted at an average age of 7.87 (SD 1.45; ranged 5‐11). Of those extracted, 36 (46.8%) were extracted before the age of 8 years and 41 (53.2%) ≥8 years. At the final evaluation, 109 premolars (52.4%) had good alignment in the dental arch and 99 (46.6%) had not. Out of 208 premolars, 128 (61.5%) were first premolars and 80 (38.5%) were second premolars. Of these, 114 (54.8%) were maxillary premolars and 94 (45.2%) were mandibular premolars.

#### 3.1.2 Analysis

Logistic regression analysis (Table 3) showed a significant difference in misalignment between Group E ≥8 and Group E <8 (*P = *.03). For Group E <8, the odds of having misalignment of the successor premolar are three times higher than for Group E ≥8. Furthermore, no difference was found between Group E ≥8 and Group E0 (*P = *.47). Also, for second premolars the odds of being misaligned are two times higher than the odds for first premolars (*P = *.02).

**Table 3:** Univariate and multiple logistic regression analysis of misalignment and associated factors

	Unadjusted OR 95% CI	*P‐*value	Adjusted OR 95% CI	*P‐*value
Extraction/exfoliation primary molar				
Group E ≥8 (ref)				
Group E < 8	2.261 0.903‐5.662	.082	2.862 1.097‐7.463	.032^a^
Group E0	1.015 0.501‐2.058	.967	1.322 0.622‐2.809	.468
Premolar				
First (ref)				
Second	1.915 1.087‐3.371	.024^a^	2.049 1.135‐3.700	.017^a^
Jaw				
Upper (ref)				
Lower	1.020 0.591‐1.763	.942	0.997 0.567‐1.755	.993
Sex				
Female (ref)				
Male	0.744 0.423‐1.307	.303	0.745 0.413‐1.343	.328

Group E ≥8 = premolars of which the predecessor primary molar was extracted when the child was 8 y or older.

Group E <8 = premolars of which the predecessor primary molar was extracted before the child was 8 y old.

Group E0 = premolars of which the predecessor primary molar has exfoliated naturally.

Abbreviations: CI, confidence interval; OR, odds ratio.

a significant

### 3.2 PUFA and DED

#### 3.2.1 Descriptive

For the analysis of DED, 29 children with 110 premolars were included (Figure 1). The 29 children (12 boys and 17 girls) had an average age of 6.93 years (SD 1.31; range 4‐9) at baseline and 9.86 years (SD 1.25; range 7‐12) when the premolars were examined.

Out of the 110 premolars, 69 (62.7%) were premolars of which the predecessor primary molar never had signs or symptoms of pulp inflammation, whereas 41 (37.3%) had a predecessor primary molar with a positive PUFA score through the course of the 3 years of follow‐up. From these, 10 presented a positive PUFA score at baseline (9.1%) and 31 (28.2%) during one of the follow‐up assessments. A total of 11 (10%) premolars presented opacities and 99 (90%) did not. Out of 110 premolars, 73 (66.4%) were first premolars and 37 (33.6%) were second premolars. Also, 64 (58.2%) were maxillary premolars and 46 (41.8%) were mandibular premolars.

#### 3.2.2 Inter‐ and intra‐examiner reproducibility

The Cohen's Kappa value for inter‐examiner reproducibility ranged from 0.77 to 0.96, whereas the intra‐examiner reproducibility ranged from 0.81 to 0.88. When the scores of ‘diffuse opacity’ and ‘sound’ were combined, in which case only distinction was made between presence or absence of ‘demarcated opacity’, the inter‐examiner reproducibility ranged from 0.86 to 1 and the intra‐examiner reproducibility ranged from 0.96 to 1.

#### 3.2.3 Analysis

Proportionally, the occurrence of DED was higher among Group *P* < 7 (21.4%), when compared to Group *P* ≥ 7 (11.1%) or Group P0 (7.2%). Logistic regression analysis (Table 4), however, showed this occurrence of DED not to be significant between the three groups (*P* > .05). Although not statistically significant, DED was proportionally more frequently observed in maxillary as compared to mandibular premolars (*P* = .06).

**Table 4:** Univariate and multiple logistic regression analysis of developmental enamel defects and associated factors

	Unadjusted OR 95% CI	*P‐*value	Adjusted OR 95% CI	*P‐*value
PUFA score primary molar				
Group *P* ≥7 (ref)				
Group *P* <7	2.182 0.378‐12.583	.383	1.124 0.169‐7.746	.904
Group P0	0.625 0.139‐2.819	.541	0.505 0.104‐2.458	.397
Premolar				
First (ref)				
Second	1.143 0.312‐4.183	.840	1.292 0.323‐5.169	.717
Jaw				
Upper (ref)				
Lower	0.120 0.15‐0.973	.047^a^	0.125 0.014‐1.082	.059
Sex				
Female (ref)				
Male	0.469 0.117‐1.872	.284	0.535 0.128‐2.239	.392

Group *P *≥7 = premolars of which the predecessor primary molar presented signs or symptoms of pulp inflammation when the child was 7 y or older.

Group *P* <7 = premolars of which the predecessor primary molar presented signs or symptoms of pulp inflammation before the child was 7 y old.

Group P0 = premolars of which the predecessor primary molar never had any signs or symptoms of pulp inflammation.

Abbreviations: CI, confidence interval; OR, odds ratio.

a significant

Our sample size for analysis of DED was limited to 110 premolars, of which only 11 showed DED. The Cramer's V revealed that the effect size was small (0.16). The ‘post hoc’ power calculation–given α (0.05), sample size (110), effect size (0.16) and degrees of freedom (2)–revealed that the power was 30%.

## 4 DISCUSSION

The aim of this study was to investigate the effect of extraction and pulp inflammation of the primary molars on their successor premolars. Outcome parameters were the (mis)alignment of the premolar in the dental arch and the presence of DED in these premolars. As brought forward in the introduction, a threshold set at the age of 8 years was considered helpful in estimating whether the misalignment of the permanent successors should be prevented in cases when the early extraction of the primary molars is required. Our results showed that misalignment of premolars occurs more frequently when the predecessor primary molars are extracted when the child is younger than 8 years old, as compared to when the extraction is performed later or in case of physiological exfoliation.

Additionally, this study showed that second premolars have a significantly higher chance of having misalignment than first premolars. This is in accordance with the literature^5^ in which it has been suggested that extraction diastemas of maxillary second primary molars spaces show the greatest space closure, followed by lower second primary molar diastemas, whereas upper and lower first primary molar diastemas show less space closure.

According to the literature,^5^, ^6^ premature extraction would lead to more loss of space in the upper than in the lower arch. Remarkably, in our study, no difference in misalignment between the upper and lower premolars was found. One explanation for this lack of difference could be the fact that, in our study, we evaluated whether the early extraction of a primary molar would result in misalignment of the successor premolar, instead of space loss in the dental arch. According to Tunison et al,^20^ evaluating the clinical implications of early extractions of primary molars is more important than just measuring the extent of space loss. Although these authors found an immediate space loss of 1.5 mm per arch side in the mandible and 1 mm in the maxilla, they discuss that although statistically significant, the magnitude of loss is of questionable clinical relevance.

Both first and second as well as upper and lower premolars were included in the present study. The literature shows that extraction of primary molars before the age of 8 years delays the eruption of permanent successors.^8^ Consequently, we have used this age as the cut‐off point for extraction of primary molars. The range of normal eruption age for premolars is 10.09 (first lower premolar) to 11.44 years old (second lower premolar).^6^ Therefore, extraction of a primary molar at the age of 8 years implies that the time to eruption of the permanent successor varies between 2 and 3.5 years dependening on which primary molar (first/second, upper/lower) has been extracted. Early extraction has a delaying effect on the eruption,^6^ the reason for which we evaluated the potential occurence of (mis)alignment.

In this study, we used the chronological age of the included children. Time of tooth eruption, however, may differ among individuals. In general, girls are ahead of boys in their dental development.^6^ The age at the onset of puberty varies with sex, generation, population, and environment and differs greatly from one person to another.^21^ A hand wrist x‐ray can be used to determine skeletal maturity^21^, ^22^, ^23^, ^24^; however because this study was performed without access to radiographic equipment, it was not possible to carry out this assessment. Literature also shows that skeletal development and dental development/ tooth eruption may vary independently.^21^, ^22^, ^23^, ^24^ Dental development/ tooth eruption can be delayed or accelerated without a corresponding change in skeletal development.^22^, ^23^, ^24^ Tooth eruption can occur several years before or after the maximum pubertal skeletal growth.^21^

Inter‐examiner reproducibility and intra‐examiner reproducibility were only calculated for DED. For (mis)alignment, this was not performed but it was chosen to score this based on consensus among examiners.

Regarding the occurrence of DED, our results showed no significant difference between Group *P* < 7, Group *P* ≥ 7, and Group P0. Interestingly, DED were also found on premolars of which the predecessor primary molars did not have a positive PUFA score. On one hand, we could attribute this finding to the multifactorial nature of these defects.^25^ On the other hand, there is a chance that some positive PUFAs may have been overlooked, as children were evaluated only every six months and without access to radiograph examination. Therefore, only clear clinical signs of pulp inflammation were recorded as positive PUFA. Furthermore, the clinical evaluations were performed by different examiners in every follow‐up assessment. All examiners, however, followed the same training and calibration exercises that were given by the same benchmark examiner. Inter‐ and intra‐examiner kappa values obtained were always above 0.7, which is considered as a substantial level of agreement.^26^ Therefore, a potential bias attributed to the different examiners was minimized.

In our study, proportionally, maxillary premolars more frequently had a DED as compared to mandibular premolars. This observation is in accordance with previous studies that found that the maxillary teeth were more commonly affected by demarcated opacities than their mandibular counterpart^4^, ^9^ and that the maxillary premolars were most often affected by demarcated opacities.^9^ This may be explained by the fact that the alveolar bone in the maxillary jaw is less dense than the bone in the mandibular jaw,^27^ which possibly eases the inflammatory process to extend towards and affect the germs of the permanent teeth.

It must be acknowledged that due to the retrospective study design, we were constrained by the variables in the data set and had to deal with heterogeneity within the groups. Some of the ‘control’ primary molars were sound, whereas others had untreated caries or were treated with ART or NRA. In an investigation by Lo et al,^9^ distinction was made between caries‐free, small caries lesions (arrested caries and restorations were coded in the same way) and large caries lesions (that were in need of pulp therapy or extraction). These authors only included decayed teeth that remained untreated during the study period, whereas in the present study some primary teeth with a positive PUFA score were extracted shortly after the diagnosis, while others were left in situ for a long time. The duration of presence of pulp inflammation could be an interesting factor to investigate, but because some positive PUFAs were already present at baseline and evaluations were only performed every six months, and a precise estimate of the duration of this inflammation process was not possible.

We acknowledge the limitation of our study related to the small sample size. Especially concerning the evaluation of DED, our sample size was limited to 110 premolars and ‘post hoc’ calculation revealed that the power was 30%. Therefore, we recommend a careful interpretation of the results regarding this outcome. The lack of statistical significance does not imply that there is no association and it might be that the proportional but non‐significant decrease in DED (when the pulp inflammation occurred after the age of 7) observed in this study would statistically benefit from a larger sample size.^28^ Nevertheless, as said before, the literature lacks on studies describing the association between the presence of pulp inflammation on a primary molar and the development of DED on the successor premolar relative to the age of the child. Reporting the results as found in our investigation is important to encourage further investigation on this important topic. Future studies following similar premises will provide useful data that combined with the present one may improve power and better clarify the association between pulp inflammation in the primary molars and developmental defects in the permanent successors.

In conclusion, the results of this study show that misalignment of premolars occurs more frequently when the predecessor primary molars are extracted before the age of 8 years, as compared to extraction performed later or in case of physiological exfoliation.

### 4.1 Why this paper is important to paediatric dentists

- Misalignment of premolars occurs more frequently when the predecessor primary molars are extracted before the age of 8 years and affect more often the second premolars.
- Interpreted with hesitation, DED on premolars might occur more frequently when the predecessor primary molars presented pulp inflammation before the age of 7 and might affect more often the maxillary premolars.
- Further investigation with larger sample sizes is needed.

## CONFLICT OF INTEREST

The authors declare that they have no conflicts of interest.

## AUTHOR CONTRIBUTIONS

FNW, CCB, and VMS conceived the ideas; FNW, GCAA, and VMS collected the data; FNW and DH analysed the data; and FNW, CCB, and DH led the writing. All authors gave final approval and agreed to be accountable for all aspects of work ensuring integrity and accuracy.
